# Computational studies of human class V alcohol dehydrogenase - the odd sibling

**DOI:** 10.1186/s12858-016-0072-y

**Published:** 2016-07-25

**Authors:** Linus J. Östberg, Bengt Persson, Jan-Olov Höög

**Affiliations:** 1 Department of Medical Biochemistry and Biophysics, Science for Life Laboratory, Karolinska Institutet, Stockholm, Sweden; 2 Department of Cell and Molecular Biology, Science for Life Laboratory, Uppsala University, Uppsala, Sweden; 3 Department of Medical Biochemistry and Biophysics, Karolinska Institutet, Stockholm, Sweden

**Keywords:** Alcohol dehydrogenase, Mutational pressure, Pseudoenzyme, Sequence analysis, Structural calculations

## Abstract

**Background:**

All known attempts to isolate and characterize mammalian class V alcohol dehydrogenase (class V ADH), a member of the large ADH protein family, at the protein level have failed. This indicates that the class V ADH protein is not stable in a non-cellular environment, which is in contrast to all other human ADH enzymes. In this report we present evidence, supported with results from computational analyses performed in combination with earlier in vitro studies, why this ADH behaves in an atypical way.

**Results:**

Using a combination of structural calculations and sequence analyses, we were able to identify local structural differences between human class V ADH and other human ADHs, including an elongated β-strands and a labile α-helix at the subunit interface region of each chain that probably disturb it. Several amino acid residues are strictly conserved in class I–IV, but altered in class V ADH. This includes a for class V ADH unique and conserved Lys51, a position directly involved in the catalytic mechanism in other ADHs, and nine other class V ADH-specific residues.

**Conclusions:**

In this study we show that there are pronounced structural changes in class V ADH as compared to other ADH enzymes. Furthermore, there is an evolutionary pressure among the mammalian class V ADHs, which for most proteins indicate that they fulfill a physiological function. We assume that class V ADH is expressed, but unable to form active dimers in a non-cellular environment, and is an atypical mammalian ADH. This is compatible with previous experimental characterization and present structural modelling. It can be considered the odd sibling of the ADH protein family and so far seems to be a pseudoenzyme with another hitherto unknown physiological function.

**Electronic supplementary material:**

The online version of this article (doi:10.1186/s12858-016-0072-y) contains supplementary material, which is available to authorized users.

## Background

Alcohol dehydrogenase (ADH:EC 1.1.1.1) is a large family of enzymes involved in the general metabolism of alcohols and aldehydes. The family members are present in a wide range of species, from bacteria to primates, where the mammalian ADHs are divided into six classes: class I–VI, whereof only class I–V are present in primates [1, 2] (Additional file 1: Table S1)^1^.

Mammalian class I–IV have been characterized extensively at DNA and protein levels where the common trait is the ability to act as a general alcohol metabolizing enzyme with NAD^+^ as electron acceptor. The substrate specificity varies between the enzyme classes, where class III ADH is the only ADH class that has been ascribed specific functions in glutathione-dependent formaldehyde scavenging and nitrosoglutathione metabolism [3].

All mammalian class I–IV ADH enzymes act as dimers, where each subunit contains two zinc atoms, one catalytic and one structural. The catalytic zinc is part of the active site, where it is coordinated by two Cys and one His [4]. However, a glutamate adjacent to the histidine has been proposed to interact in the zinc coordination in class III ADH [5]. The structural zinc is located in a loop at the edge of the enzyme where it is coordinated by four Cys and has been shown to be crucial for the structure and dimer formation [4, 6].

Class V ADH was first identified at the DNA level in humans and deer mouse [7, 8]. Originally, this human ADH was believed to lack the ninth (last) exon due to a splice variant, but transcripts containing all nine exons were later discovered [9]. In humans, the transcripts have been found primarily in liver and, with lower expression levels, in duodenum and kidney ([10], Human Protein Atlas [11] accession: ADH6)^1^, while the corresponding rat transcripts have been detected mainly in kidney, with lower expression also observed in liver, stomach, duodenum and colon [12]. Class V ADH has never been isolated nor identified in any tissue at the protein level, though peptides mapped to the protein have been identified in recent proteomics experiments (PeptideAtlas [13] accession: P28332), implying that the protein is indeed expressed. Several research groups have attempted to express the protein in vitro, but to date class V ADH has been expressed only as a fusion protein with green fluorescent protein (GFP) and glutathione-S-transferase (GST), albeit with no enzyme activity traced [12]. Human class V ADH enzymatic properties were once reported [14], but so far without confirmation from any other research group.

The class V ADH sequences that are available in the databases are predicted from genome assemblies, with the human, deer-mouse, and rat sequences primarily obtained from cDNA analyses. Of the genes corresponding to class V ADH that have been analyzed so far, only the mouse gene has been annotated as a pseudogene as the gene lacks multiple exons. The latter has been confirmed after multiple revisions of the genome sequence (GenBank: NR_033581). In addition, the mouse genome contains two genes corresponding to class VI ADH, which is true for all analyzed rodents.

Class VI ADH is only confirmed at the transcript level and is not present in humans. Class V ADH and class VI ADH were originally believed to belong to the same class, but with the analysis of an increasing number of genomes, it has become increasingly evident that class VI ADH forms a class of its own [15]. As a result of this, a gene called ADH6 in a database may correspond to either class V or class VI ADH. The sequence identity between the classes is the usual 60 %, and the classes can easily be separated using e.g. class-specific HMM profiles.

Due to the lack of identification of actual proteins for class V ADH, it is believed that this ADH class may not have any traditional ADH activity, or potentially is a pseudoenzyme, as only peptide fragments have been detected [15]. Where traditional biochemical analyses fail to determine functional and structural characteristics, computational approaches constitute valuable alternatives that can calculate the protein properties, based upon knowledge of its components and their physico-chemical characteristics. One example is molecular dynamics (MD) calculations utilized to investigate the stability of a protein [16] which is difficult to analyze with other techniques.

In the current study, class V ADH, with focus on the human form, has been investigated at the DNA and protein levels using computational methods to explain the lack of success in the isolation and detection of functional class V ADH proteins.

## Methods

### Structure comparisons

The crystal structure of human class Iγ ADH 1u3w [17]) was obtained from the RCSB PDB, www.rcsb.org/pdb/, and used as template to create homology models of class III ADH and class V ADH with the ICM 3.7 software (MolSoft LLC, LaJolla) [18, 19]. The full nine-exon sequence of the human class V ADH was used, corresponding to isoform 2 in UniProt (P28332). The model generation followed the standard ICM procedure with minor modifications to allow for the inclusion of the two zinc atoms and NADH at the active site of each chain and for dimer formation. The model of human class III ADH was used as a control to confirm the accuracy of the methods and as a baseline for comparisons. The sequence identities between the template and the class III ADH and class V ADH are in the 58–62 % range. The modelling accuracy was evaluated using the TM-score (template modelling score) server [20]. For an overall investigation of the reliability of the method, a modelling using class II, III, and IV ADH as templates (3cos, 2fzw, and 1d1t) was also performed. The obtained results were very similar to those based on class I ADH, and are thus not discussed here.

The structural stability was evaluated by performing MD simulations using GROMACS 4.6 with the G53a6 force field. Each model was energy minimized, followed by triplicate runs of equilibration through a 100 ps NVT (Number of particles, Volume, and Temperature maintained at a constant level) and a 100 ps NPT (Number of particles, Pressure, and Temperature maintained at a constant level) ensemble, finally followed by 20 ns production runs. The temperature and pressure were maintained (300 K and 1 bar, respectively) using the V-rescale and Parinello-Rahman approaches [21–28]. The full set of parameters is available in the Swedish bioinformatics infrastructure repository (DOI: 10.17044/BILS/p000002).

The deprotonated cysteines involved in the binding of the zinc atoms (“CYM”) were defined as normal “CYS” residues with a charge of –0.800 on the sulphur atom (SG) and –0.200 on the adjacent carbon atom (CG).

Structural analysis was performed using the tools included in GROMACS and graphical analysis of the structures was performed using ICM 3.7 and Pymol 1.7 (Schrödinger, LLC). Secondary structure assignment was performed using the DSSP software [29].

A general screening for the structural properties of the dimerization region identified during the other experiments was also performed. All ADH crystal structures associated with the ADH_N and ADH_zinc_N entries in Pfam [30] were obtained from the RCSB PDB. Non-dimeric structures or structures with incorrect subunit orientations were removed. The amino acid sequences were aligned using MAFFT and the structures were evaluated using DSSP. The alignment was used to identify the relevant residues in each of the structures and the relevant features were extracted from the DSSP output.

### Unique residues among the class V ADH sequences

A total of 597 unique mammalian class I–VI ADH protein sequences were obtained from the UniProt, NCBI protein, and Ensembl databases in October 2015 using a previously described methodology [15]. All sequences were aligned using the L-INS-i approach of MAFFT 7.266 [31, 32].

The alignment was then analyzed on a per-class basis. Positions containing no residues from any sequences belonging to the currently analyzed class were removed, followed by a division into two parts, one containing all sequences from the class, the other containing all the other sequences. For each class alignment a position-specific scoring matrix (PSSM) was calculated, using the residue ratios as scores. Finally, a scoring function was used on each position in the alignment:

$$ s(pos)=\left\{\begin{array}{c}\hfill PSS{M}_{rest}{(pos)}_{aa}>0:\frac{ \max {\left(PSS{M}_{class}(pos)\right)}^3}{PSS{M}_{rest}{(pos)}_{aa}}\hfill \\ {}\hfill PSS{M}_{rest}{(pos)}_{aa}=0:\frac{ \max {\left(PSS{M}_{class}(pos)\right)}^3}{\frac{1}{seq{s}_{rest}}/2}\hfill \end{array}\right. $$

max*(PSSM*_*class*_*(pos))* is the highest conservation rate at a position for the studied class and *PSSM*_*rest*_*(pos)*_*aa*_*is* the conservation rate of the same residue type at the same position (fulfilling max*(PSSM*_*class*_*(pos))*) among the other classes*.* The second case is used to avoid division by zero when the residue type is not observed in the other classes, *seqs*_*rest*_ being the number of sequences among the other classes.

As the scoring function gave a high score to positions with a high level of conservation among the studied class and a low prevalence of the conserved residue in other classes, the results were used in order to identify positions worth investigation among the class V ADH sequences.

### Intra-class protein sequence variation

Intra-class sequence variation was determined for the ten species who have at least one sequence determined from each of the six ADH classes (Brandt’s bat, Chinese hamster, Chinese tree shrew, cow, deer mouse, little brown bat, prairie vole, rat, water buffalo, and yak; primates not included due to the lack of class VI ADH). The sequences of these proteins were acquired from the UniProt and NCBI Protein databases in October 2015 All sequences of each class were aligned using the L-INS-i approach of MAFFT 7.266 [31, 32], giving a total of six multiple sequence alignments. Each of the sequences was compared pairwise with all the others in the same alignment, calculating a sequence identity percentage for each comparison. The alignments were trimmed to remove positions only containing residues from a few sequences (e.g. trailing ends). The sequence identities were then compared and the median and mean sequence identities were calculated.

### Phylogeny/evolutionary analysis

The mRNA/cDNA sequences corresponding to the above set of proteins of all class I–VI ADH proteins available in NCBI Protein as of October 2015 were retrieved. Protein entries without linked cDNA/mRNA (spliced nucleotide sequences) were not included among the final nucleotide sequences. This gave a total of 416 ADH nucleotide sequences.

The sequences were aligned using the L-INS-i approach of MAFFT 7.266. The resulting alignments were manually curated to confirm that the final alignment included the coding sequences in the correct reading frames. Further curation was performed by removing all sequences with a high amount of gaps in the aligned coding region, reducing the amount of sequences to 114 class I ADH, 53 class II ADH, 72 class III ADH, 60 class IV ADH, 39 class V ADH, and 49 class VI ADH nucleotide sequences.

A phylogenetic tree was generated for each class using the default parameters of Phyml release 20151210 [33]. The tree was used as input for PAML 4.8a [34] to perform an evolutionary analysis of the sequences to evaluate whether there was an evolutionary pressure for the development of the sequences using the ratio of non-synonymous versus synonymous mutations, dN/dS, for which values below 1 indicate an evolutionary pressure [35]. A reference phylogenetic tree for all known mammalian ADH protein sequences was also generated using MAFFT and PhyML.

## Results

Two structural models, one of human class V ADH and one of human class III ADH, were generated using the RCSB PDB entry 1u3w (class Iγ ADH/ADH1C) as template. Both models had a high similarity to their template (RMSD < 1 Å). The TM-score was 0.76 for the model of class III ADH (compared to the crystal structure 2fzw). Further, MD simulations were used to refine these models and to evaluate their structural properties over time. The simulations converged after ca. 1 ns at an RMSD of ca. 2.5 Å.

**Fig. 1:**
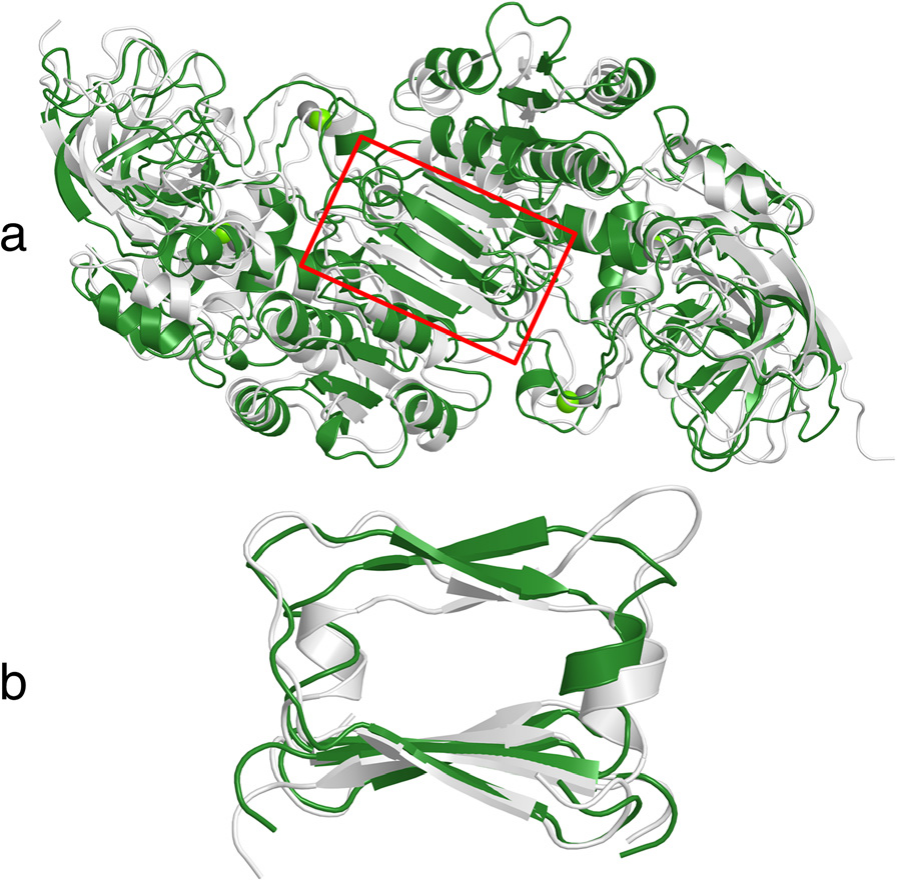
Comparison of dimers of class Iγ ADH and class V ADH after 20 ns of molecular dynamics simulations. The enzyme models of class Iγ ADH (*white*) and class V ADH (*green*) have a high level of structural similarity. **a**: The full dimers of class Iγ ADH and class V ADH after 20 ns of molecular dynamics simulations. **b**: The dimer interaction region (position 282–320) marked in *red* in A, rotated 90°. It contains a short β-α motif. The α-helix was not identified by DSSP after two out of three molecular dynamics simulations of the class V ADH model; one such run, with non-α-helical Ramachandran angles, is shown here. This tendency was not observed in any other ADH models (e.g. class Iγ ADH, *white*), and only in mouse class II ADH among the structures from multicellar organisms available in the RCSB PDB

No general structural instability could be observed in the class V ADH model during the simulated time period of 20 ns. However, it was observed that the class V ADH model formed elongated β-strands (five residues in class V ADH, versus three residues in the reference model of class III ADH), one β-strand in each chain, in the region involved in dimer interaction. This was not observed in the reference class III ADH model, or in the template crystal structure of class Iγ ADH, neither before nor after MD simulations. A comparison of the model structures of class III ADH and class V ADH after the MD simulation showed additional differences at the dimer interface (Fig. 1). One of the two α-helices located next to the elongated β-strands (one in each subunit) was no longer identified by using the DSSP software after the MD simulations in two out of the three class V ADH simulations, implying the unfolding of that structural element. Further investigation, including Ramachandran plots, indicated that the lack of identification was due to the nearby glycines being forced into unfavourable φ and ψ angles, and in one case also due to large atomic distances (>5 Å), preventing the formation of hydrogen bonds (Fig. 1). In contrast, both α-helices at each side of the β-strands were correctly identified by using DSSP for all reference model simulations.

The aforementioned α-helix was identified in all ADH crystal structures found in the RCSB PDB by using DSSP. A 3-residue β-strand was observed in 75 out of 106 ADH structures while 25 contained a 5-residue β-strand, three of which were from a multicellular organism (three conformations of mouse class II ADH). One structure contained no β-strand, one contained a 2-residue β-strand, and four contained a 4-residue β-strand. Thus, most mammalian ADHs have a 3-residue β-strand in their dimer interaction regions, implying that the 5-residue β-strands observed in our class V ADH simulations are rare.

**Fig. 2:**
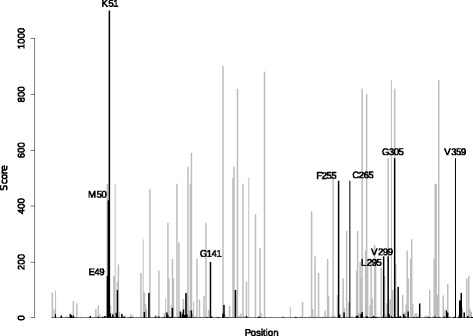
Conserved amino acid residues in class V ADH with a low prevalence in other ADH classes. The scoring function represents the highest rate of conservation for one residue type at one position in one class compared to the conservation ratio for the same residue type at the same position in the other classes. The labelled peaks represent the position and residue type seen in human class V ADH. *Black* peaks represent class V ADH and the *grey* peaks represent class I–IV ADH

**Fig. 3:**
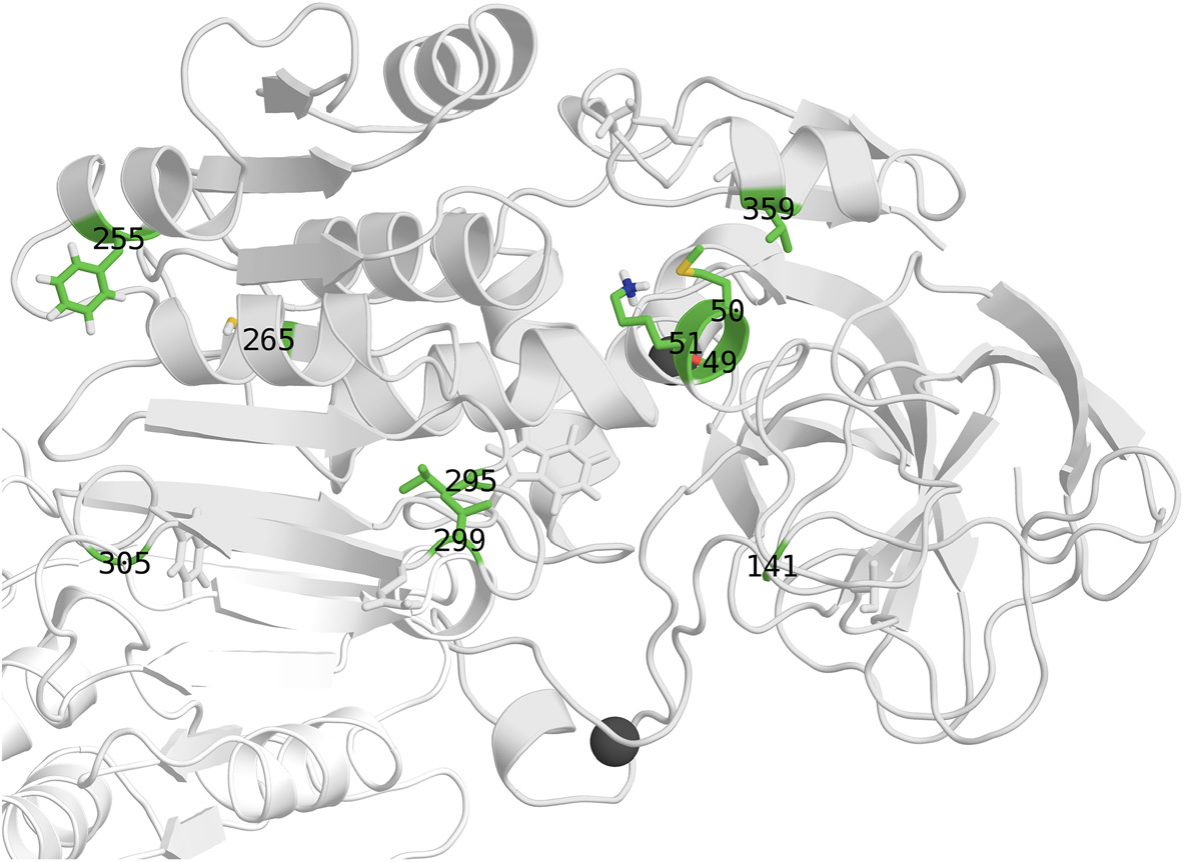
Unique residues in class V ADH. The residues listed in Table 1 highlighted on a monomer from a model of class V ADH based on 1u3w after 20 ns of molecular dynamics simulations. *White*: one subunit of class V ADH, *green*: the unique residues listed in Table 1, *dark grey*: Zn^2+^

Sequence analysis was performed to compare the positional conservation rates of class V ADH to those of the other classes using a PSSM-based method. This identified several positions of interest (Table 1), visualized as labelled peaks in a schematic representation of the sequence (Fig. 2) and in a model of class V ADH (Fig. 3). The unique residues that were identified include those located at or near the catalytic site (Glu49, Met50, Lys51, Gly141, Val359), the dimer interaction region (Leu295, Val299, Gly305), the outer active site (Phe255), and inside a central structural element (Cys265).

**Table 1:** Unique residues in class V ADH

Residue	Protein localization	Prevalence class V	Prevalence other classes	Residues in other classes
Glu49	Active site	>80 %	<1 %	Asp in other classes (>95 %).
Met50	Active site	>70 %	0 %	Asp in class I and IV (>85 %), Ala in class II and III (>95 %), Asp or Asn in class VI (>70 %).
Lys51	Active site	100 %	0 %	His in class I and IV (>90 %), His or Thr in class II, Tyr in class III (>99 %), His in class VI (100 %)
Gly141	Active site	>55 %	0 %	Leu in class I (>65 %), Met in class II (>55 %), Met in class III (>95 %), Met in class IV (>80 %), Phe in class VI (>65 %)
Phe255^a^	Surface	>75 %	0 %	Lys in class I (>60 %), Ile in class II and III (>60 %), Ser in class IV (>90 %), Met or Val in class VI (>65 %)
Arg265	In central β-sheet	>75 %	0 %	Ser in class I and III (>95 %), Ala in class II (>80 %), Thr in class IV (>75 %), Ala in class VI (>90 %)
Leu295	Dimer interaction	>55 %	0 %	Pro in class I (>90 %), Ala in class II (>45 %), Ala in class III (>90 %), Pro in class IV (100 %), Ala in class VI (>80 %)
Val299	Dimer interaction	>55 %	0 %	Gln in class I (>90 %), Lys in class II (>50 %), Glu in class III (>90 %), Lys in class IV (>90 %), Ser in class VI (>65 %)
Gly305	Dimer interaction	>80 %	0 %	Pro in others (class I >99 %, class II >65 %, class III >95 %, class IV > 95 %), Ala or Pro in class VI (>98 %)
Val359	Near active site	>80 %	0 %	Phe in others (class I >99 %, class II >75 %, class III >95 %, class IV >95 %, class VI >70 %)

^a^This position contains Cys in human class V ADH, but Arg is the most common residue among mammalian class V ADHs

Positional numbering according to the human class V ADH protein, Ser1 being the first residue

In order to estimate the variation of the members within each class, the intra-class sequence identities were calculated (Table 2). Class V ADH has the largest mean intra-class variation, but the variation of class II ADH and class VI ADH are at similar levels.

**Table 2:** Pairwise intra-class sequence identities within each of the six mammalian ADH classes

ADH class	Median	Mean	Min	dN/dS
I	82.5 %	84.2 %	78.2 %	0.308
II	73.7 %	76.6 %	63.7 %	0.332
III	92.5 %	92.8 %	86.9 %	0.126
IV	83.7 %	81.5 %	68.7 %	0.228
V	70.0 %	75.4 %	64.5 %	0.385
VI	76.9 %	75.9 %	64.5 %	0.288

Calculated from the sequences of the ten species (Brandt’s bat, Chinese hamster, Chinese tree shrew, cow, deer mouse, little brown bat, prairie vole, rat, water buffalo, yak) that have at least one identified member in each of the classes

The mammalian ADH enzymes were also analyzed with respect to synonymous (dS) and non-synonymous (dN) nucleotide substitutions to estimate the evolutionary pressure on the proteins, where a ratio dN/dS less than 1 implies the existence of an evolutionary pressure. The strongest evolutionary pressure (dN/dS) was determined for class III ADH, followed by class IV ADH and class I ADH, while the lowest evolutionary pressure was observed for class II ADH and class V ADH. For comparison, we also performed the same analysis for the mammalian members of the well-conserved Histone H1 and the quite variable Interleukin-2, resulting in ratios of 0.055 and 0.556, respectively.

**Fig. 4:**
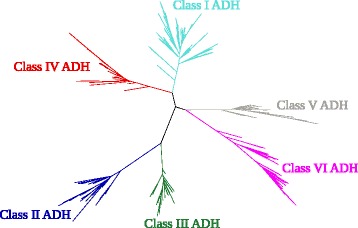
Phylogenetic tree of all known mammalian ADH protein sequences available in the major databases as of October 2015. The six mammalian ADH classes are easily identified

A reference tree of all known mammalian ADH proteins was generated, showing the relations between the six ADH classes (Fig. 4).

## Discussion

In order to investigate the class V ADH characteristics, we performed structural calculations. These methods, including MD simulations, have strength in giving insight into overall and local structural properties of proteins where, for different reasons, it is not possible to utilize traditional biochemical analyses. For human class I–IV ADH, and the corresponding structures from other mammals, all available crystal structures show a very high level of overall similarity. It is therefore not surprising that the generated models of human class III ADH, and class V ADH are very similar both when compared to their templates and when compared to each other. The TM-score of the comparison reference model (class III ADH) and its crystal structure (2fzw) was 0.76, implying that the modelling method is reliable for ADH structures.

However, after 20 ns of MD simulations, each chain of the class V ADH models formed an elongated β-strand in the dimerization region and the adjacent α-helix was destabilized in two out of three simulations (Fig. 1), the elongated β-strand matching pilot investigations done on the rat class V ADH [12]. These structural elements are located in the region responsible for most of the subunit interactions (positions 290–320). It should be stressed that this was observed rarely in the structures of class I–IV ADH from any species. It was not observed in the reference models, and only in crystal structures of ADHs from single-cellular organisms and in mouse class II ADH. The latter is known to be inactive due to a mutation in another part of the enzyme, which could allow for further alterations [36]. Elongated β-strands have also been observed in the tetrameric structure of yeast ADH [37], which has the 5-residue β-strand observed in class V ADH. However, the tetrameric formation is probably promoted by a 21-residue deletion in the region of the structural zinc which is not present in class V ADH, and thus probably not relevant to human class V ADH.

The formation of dimers has been shown to be essential for the stability of mammalian ADH enzymes [4], and a monomeric ADH enzyme has only been reported in one study [38]. The elongated β-strands and the unfolded α-helices in this region imply that there are structural changes occurring at the dimerization region. The Ramachandran plots for the α-helices denote that Gly located at the start of the helices are forced into unfavoured conformations, which in turn implies that the simulations are attempting to make large changes to the structure.

The observed changes in the dimer formation region could be due to the alteration of a highly conserved Pro in class I–IV ADH to Gly in class V ADH. The Pro would be a clear divider between the β-strand and the α-helix, and could also support the formation of the α-helix, while the Gly would allow either type of secondary structure, elongating one and causing instability in the other. The unfolding of the α-helix could also be the result of the simulation compensating for structural bias caused by the homology modelling approach, where the initial model forced the protein into a slightly unfavoured conformation. The simulation could then be forced to compensate for the bias by forcing e.g. the Gly into an unfavoured conformation, explaining the Ramachandran outliers.

These observations, the elongation of the β-strands and the the instability of the α-helix, no matter if it is a real observation or caused by modelling bias, give a strong signal that the class V ADH structures have structural alterations at the dimer formation region. Even though this region has a varying set of residues between class I–IV, it has a very high structural conservation, and any alterations there could alter the stability of the rest of the structure [4, 6].

Class V ADH shows several differences in the conservation of certain amino acid residues when compared to other ADH enzymes (Table 1), which probably can explain some of the atypical behaviour of class V ADH.

Glu49, Met50, and Lys51 are located at the catalytic site. In class I and class IV ADH, this triplet is usually Asp-Asp-His, in class II the most common triplet is Asp-Ala-His, and in class III Asp-Ala-Tyr is almost perfectly conserved. Most sequences among class I–IV ADH have an aromatic residue (His/Tyr) at the third position (position 51 in Table 1), the only exception being a few class II ADH sequences with Thr instead. It has been shown that this position is involved both in the binding of the co-enzyme (NAD^+^) and the actual catalytic reaction [4]. The size of Lys, along with the lack of an aromatic ring, makes this notable, as it could lead to a major alteration of the enzymatic activity or extinguish it as such. It should also be noted that this position is perfectly conserved within the class V ADH class, implying a specific function.

Cys265 (Cys in human class V, but Arg is the most common among the class V ADH enzymes; both are only present in class V ADH) is part of the central β-sheet. The position is class-unique with a high level of conservation within each class. The human residue, Cys, shares some properties with the residue types found in other classes (Ala/Ser/Thr), as opposed to the Arg that has a much higher level of conservation among the class V ADH proteins.

Leu295, Val299, and Gly305 are located in the region where most dimer interactions occur. Leu295 is located in the loop connecting the central β-sheet with the loop at the interaction site. The position is weakly conserved in class V ADH, where Ala and Pro are the most common residues among the other ADH classes. Val299 is one residue upchain of the elongated β-strand observed during the MD simulations. The residue is class-specific, with Gln, Lys, Glu, and Lys being the most common residue types in class I, class II, class III, and class IV, respectively. Gly305 is the class V-specific replacement of the fairly well-conserved Pro in class I–IV. It is located at the late turn of the loop in the dimer interaction region, one position from the short α-helix present in class I–IV. It may be involved in the structural alterations at the dimerization region.

The amino acid sequence of class V ADH shows ca. 60 % positional identity to those of the other ADHs, similar to that of the inter-class differences between any other ADH classes. For sequences analyzed in this study, the median and mean intra-class sequence identities among the class V ADH sequences are the lowest observed for any class; 70 and 75 % (Table 2), but the levels are in the vicinity of those of other classes, e.g. class II ADH: 74 and 77 %, respectively. While there is a variation among the species represented within each class, there is a slightly lower intra-class similarity between the class V ADH sequences than among the other classes.

This tendency could also be observed when calculating the evolutionary pressure, dN/dS [35], on the different ADH enzymes. class III had the strongest evolutionary pressure with a dN/dS ratio of 0.126, followed by class IV, class I, class II, and finally class V at 0.385, placing the class V ADH dN/dS ratio higher than that of many other proteins, but far below the level of some highly variably proteins with both specific and important functions.

Class III ADH is known to be the ancestral form of the zinc-containing ADHs [2] and shows a crucial function in glutathione dependent formaldehyde scavenging as well as in NO metabolism. The enzyme has been detected, so far, in all species harbouring glutathione from bacteria to primates [3], and as such, a high evolutionary pressure was expected.

The other well-characterized ADHs are involved in general alcohol dehydrogenase activities, where the class I ADH enzymes tend to have a general detoxifying function, aiding in the metabolism of many alcohols including ethanol and hydroxysteroids. Class II ADH is believed to be involved in the metabolism of retinols and aromatic alcohols, e.g. hydroquinone. Worth noting is that some rodent species have a Pro at position 47, rendering their enzymatic activity near non-existent [36]. Finally, class IV is the main ADH involved in the retinoid metabolism, e.g. in cell differentiation [39], and in addition in the first-pass ethanol metabolism in humans [40].

Class V ADH can be expressed under certain conditions, as shown from Northern blot, in vitro translation and proteomics analyses [11, 12]. However, a stable and active protein has never been reported. Fused recombinant class V ADH has been expressed both as GFP-class V ADH and GST-class V ADH. In none of these cases any activity with known substrate for ADHs was traced [11], where other mammalian ADHs under similar conditions showed activity. For other protein families, pseudoenzymes have been ascribed as integral parts of the cell system, where they today play unknown roles [41].

Based on the functions of the enzymes, it can be concluded that the dN/dS ratios make sense, the enzymatic specificity having a correlation to the level of evolutionary pressure [35]. As e.g. class II ADH has a lower dN/dS ratio than class V ADH, even with some species having a common variant with near no activity, it implies that some (or all) species may lack activity in class V ADH as well. However, as the calculated value for class V ADH was still much lower than 1 (0.385), it indicates that there is an evolutionary pressure on the class V ADH sequence with a bias towards eliminating non-functional mutations, and class V ADH thereby has a supposed, but currently unknown, function in humans.

The phylogenetic tree shows that class V and class VI ADH are unique and separate classes that should not be mixed up, confirming earlier results [15].

## Conclusions

The gene corresponding to class V ADH is mainly expressed in the liver, and peptide fragments matching the protein has been detected in mass spectrometry experiments. The protein has never been isolated, but it has been expressed as fusion proteins with GST and GFP, but with no trace of the expected dimers, nor any enzyme activity with the traditional ADH substrates.

Due to the lack of success in isolating the protein, class V ADH has here been investigated using computational methods. MD simulations observed structural irregularities in the region responsible for dimer formation, which is known to be very important for the stability of the other mammalian ADH enzymes. Further, we have observed many positions that are strongly conserved in class I–IV ADH, but have in class V ADH been replaced with another amino acid residue. These observations show that there are structural irregularities in class V ADH as compared to the other mammalian ADH enzymes. At the same time, the fact that there is an evolutionary pressure among the known class V ADH sequences, together with the fact that segments of class V ADH has been observed as peptides imply that the protein is expressed.

Class V ADH is postulated to be expressed, but unable to form dimers, and thus to behave as a dead enzyme with very different properties than other ADH enzymes. The conclusions from the structural calculations are supported by the previous experimental studies [12]. Similarly, the metabolically related aldehyde dehydrogenase family harbours several dead enzymes [42].

Class V ADH can thus be considered the odd sibling of the ADH enzyme family, with multiple unique residues and structural properties not observed in other ADH proteins, and so far seems to be a pseudoenzyme, albeit it may have another hitherto unknown physiological function.

## Abbreviations

ADH, alcohol dehydrogenase; GFP, green fluorescent protein; GST, glutathione-S-transferase; MD, molecular dynamics

## Additional file

Additional file 1: Table S1. Nomenclature for Human Alcohol Dehydrogenase. (PDF 36 kb)

## Footnotes

1 The genes were originally named after the order they were discovered in each species, but as this paper is focused on the proteins, it uses the class system. This means that some protein names in the databases may deviate from the names used here. A nomenclature was proposed in 1999 [43], attempting to join the protein and gene nomenclatures. The human ADH naming is summarized in Additional file 1: Table S1.
