# Supplementary material for: Computational studies of human class V alcohol dehydrogenase - the odd sibling
Source: BMC Biochem. 2016 Jul 25;17:16. doi: 10.1186/s12858-016-0072-y (PMC4960878; doi:10.1186/s12858-016-0072-y)
Supplement: Additional file 1: Table S1. — Nomenclature for Human Alcohol Dehydrogenase. (PDF 36 kb) [file 12858_2016_72_MOESM1_ESM.pdf]

Supplementary Table 1: Nomenclature for Human Alcohol Dehydrogenase

The Class System		Suggested System Protein and Gene	Uni-Prot KB	Gene Nomenclature New	Old
Class I	α-subunit	ADH1A	ADH1A_HUMAN	ADH1A	ADH1
Class I	β-subunit	ADH1B	ADH1B_HUMAN	ADH1B	ADH2
Class I	γ-subunit	ADH1C	ADH1G_HUMAN	ADH1C	ADH3
Class II	π-subunit	ADH2	ADH4_HUMAN	ADH4	ADH4
Class III	χ-subunit	ADH3	ADHX_HUMAN	ADH5	ADH5
Class IV	μ/σ-subunit	ADH4	ADH7_HUMAN	ADH7	ADH7
Class V		ADH5	ADH6_HUMAN	ADH6	ADH6
